# A role for the vitamin D pathway in non-intestinal lesions in genetic and carcinogen models of colorectal cancer and in familial adenomatous polyposis

**DOI:** 10.18632/oncotarget.12768

**Published:** 2016-10-19

**Authors:** Yong-Sik Bong, Shahin Assefnia, Therese Tuohy, Deborah W Neklason, Randall W Burt, Jaeil Ahn, Paul J Bueno De Mesquita, Stephen W. Byers

**Affiliations:** ^1^ Georgetown-Lombardi Comprehensive Cancer Center, Department of Oncology, Georgetown University School of Medicine, Washington, DC, United States of America; ^2^ Huntsman Cancer Institute, University of Utah, Salt Lake City, UT, United States of America; ^3^ Department of Biostatistics, Bioinformatics and Biomathematics, Georgetown University School of Medicine, Washington, DC, United States of America

**Keywords:** gardner's syndrome, anal cancer, vitamin D receptor, azoxymethane, colon cancer

## Abstract

Vitamin D is implicated in the etiology of cancers of the gastrointestinal tract, usually characterized by alteration in the APC/β-catenin/TCF tumor suppressor pathway. The vitamin D receptor (VDR) is also implicated in cardiovascular and skin diseases as well as in immunity. Activated VDR can indirectly alter β-catenin nuclear localization and directly suppress β-catenin/TCF mediated transcriptional activity. We treated VDR null mice with the carcinogen azoxymethane (AOM) and generated mice bearing a mutated APC (hypomorph) on a VDR null background (*Apc^1638N/+^Vdr^−/−^*). VDR null mice do not develop GI or extra-colonic tumors but loss of VDR decreased intestinal tumor latency and increased progression to adenocarcinoma in both models. AOM treatment of VDR null mice also caused squamous cell carcinoma of the anus. Although levels and distribution of total or activated β-catenin in the epithelial component of tumors were unaffected by loss of VDR, β-catenin dependent cyclin D1 expression was affected suggesting a direct VDR effect on β-catenin co-activator activity. Extra-colonic mucosa manifestations in *Apc^1638N/+^Vdr^−/−^* animals included increased nuclear β-catenin in submucosal stromal cells, spleno- and cardiomegaly and large epidermoid cysts characteristic of the FAP variant, Gardner's syndrome. Consistent with this, SNPs in the VDR, vitamin D binding protein and CYP24 as well as mutations in APC distal to codon 850 were strongly associated with Gardners syndrome in a cohort of 457 FAP patients, This work suggests that alterations in the vitamin D/VDR axis are important in Gardner's syndrome, as well as in the etiology of anal cancer.

## INTRODUCTION

Familial adenomatous polyposis (FAP) is an inherited colorectal cancer syndrome that is classically characterized by the early onset of hundreds to thousands of adenomas in the rectum and colon [1]. Left untreated at an early stage, nearly 100% of people with FAP will develop colorectal cancer. FAP is a highly penetrant autosomal dominant disease that results from a germline mutation in the adenomatous polyposis coli (APC) gene located on the chromosome 5 [2, 3]. APC is a tumor suppressor protein that plays a critical role in Wnt signaling in part by regulating the degradation and distribution of β-catenin. In a typical canonical Wnt/β-catenin signaling pathway, a destruction complex consisting of APC, GSK3β (glycogen synthase kinase 3β), CK-Iα (casein kinase-Iβ) and AXIN induces phosphorylation of β-catenin at Ser33, Ser37, Thr41 and Ser45, respectively and then targets the phosphorylated β-catenin for proteasomal degradation by the 26S proteasome complex [4]. However, in the presence of active Wnt ligands or mutation of APC, the destruction complex cannot function properly in mediating β-catenin phosphorylation, resulting in the accumulation of β-catenin in the cytosol, which then enters the nucleus where it forms a complex with members of T cell factor/lymphoid enhancer factor (TCF/LEF) family transcription factors, altering the expression of various genes affecting the proliferation, differentiation, migration, and apoptosis [5]. Beyond the occurrence of colorectal adenoma, many FAP patients develop various extracolonic manifestations, including epidermoid cysts, desmoid tumor, osteomas, lipomas and congenital hypertrophy of the retinal pigment epithelium (CHRPE) [6, 7]. In particular, Gardner's syndrome, a variant of FAP, is characterized by particularly rapid colon cancer progression, extra-colonic manifestations such as large epidermoid cysts, and is considered more life threatening [8]. In some studies, the Gardner's Syndrome sub-type of FAP is associated with severely truncated forms of APC [9]. However the bone defects and multiple skin lesions that characterize Gardner's syndrome suggest that alterations in the function of vitamin D pathway genes together with APC mutation may play a role in this deadly disease [10–12]. As an essential regulator for calcium and bone homeostasis, vitamin D3 plays an important role in the prevention of nutritional rickets, a defect in bone development due to inadequate uptake of dietary calcium [13]. In addition, epidemiological studies point to alterations in active vitamin D levels being responsible for the protective effects of sun light exposure on the incidence of several cancers, particularly those of the colon [14, 15]. Furthermore, the most active form of vitamin D (1, 25-dihydroxyvitamin D3) inhibits cell proliferation, induces differentiation of human colon cancer cells by regulation of E-cadherin expression and by sequestrating the formation of transcriptionally active β-catenin/TCF complex through the direct binding of VDR to β-catenin [16–20]. Taken together these studies indicate that variation of vitamin D itself or vitamin D pathway genes may not only affect colorectal cancer development initiated by activated β-catenin signaling, but may also associate with the occurrence of extracolonic manifestations in FAP patients. In the present work we used carcinogen and genetic models to show that in addition to accelerating gastrointestinal tumor progression somatic loss of the VDR results in extra-colonic lesions that model the Gardner's syndrome form of FAP (on an APC mutant background) and anal cancer (following carcinogen administration). We go on to demonstrate an association between extracolonic lesions, polymorphisms of vitamin D pathway genes and site of APC mutation in a cohort of 457 FAP patients.

## RESULTS

### Loss of VDR accelerates colon cancer progression in the *Apc^1638N/+^ mouse*

**Figure 1:**
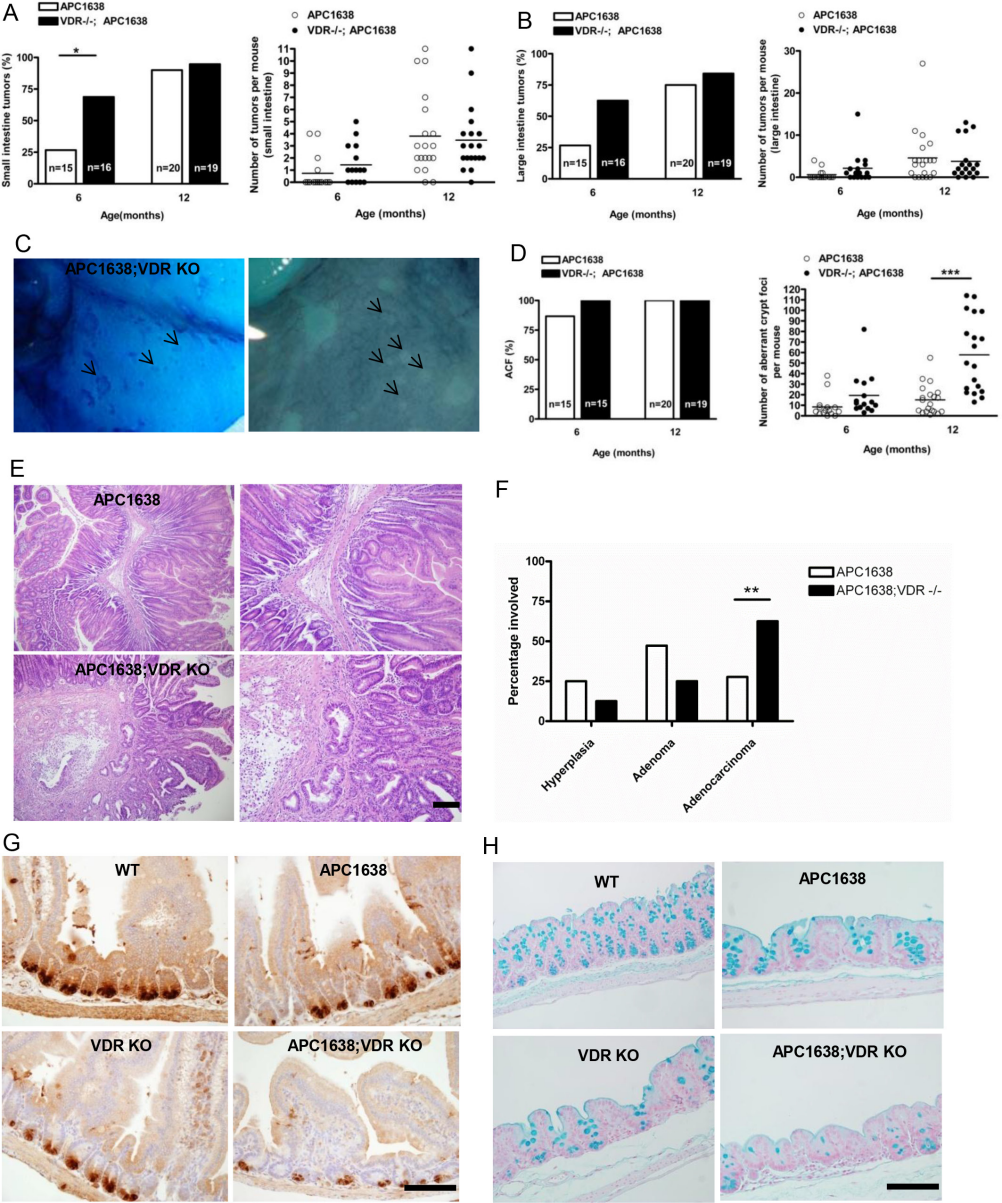
Loss of VDR does not affect tumor incidence and multiplicity but significantly increases aberrant crypt foci in the intestine of *Apc^1638N/+^Vdr^−/−^* mice (**A**) Comparison of small intestinal tumor incidence (left) and multiplicity (right) between *Apc^**1638N**^*^/+^ (*n* = 15 at 6 months 0.7333 ± 0.372, *n* = 20 at 12 months 3.8 ± 0.7384 and *Apc^**1638N**^*^/+^*Vdr*^−***/***−^ (*n* = 16 at 6 months 1.438 ± 0.3870, *n* = 19 at 12 months 3.474 ± 0.6277) mice. Each dot corresponds to one mouse and the horizontal line indicates the mean. *P < 0.032, Student's test. (**B**) Comparison of large intestinal tumor incidence (left) and multiplicity (right) between *Apc^**1638N**^*^/+^ (*n* = 15 at 6 months 0.6 ± 0.3207, *n* = 20 at 12 months 4.6 ± 1 .396 and *Apc^**1638N**^*^/+^*Vdr*^−***/***−^ (*n* = 16 at 6 months 2.125 ± 0.9259, *n* = 19 at 12 months 3.789 ± 1.005) mice. Each dot corresponds to one mouse and the horizontal line indicates the mean. (**C**) Methylene blue staining of aberrant crypt foci (ACF) in proximal colon of *Apc^**1638N**/+^Vdr*^−***/***−^ mice. (**D**) Comparison of ACF incidence (left) and multiplicity (right) between *Apc^**1638N**^*^/+^ (*n* = 15 at 6 months, 8.467± 2.811, n = 20 at 12 months, 15.20 ± 3.115) and *Apc^**1638**N/+^Vdr*^−***/***−^ (*n* = 15 at 6 months 19.33 ± 5.2, *n* = 19 at 12 months 57.84 ± 8.144), ***P < 0.0001, Student's test. Each dot (right) represents one mouse and the horizontal line indicates the mean. (**E**) Representative hematoxylin/eosin staining images of intestinal tumors from *Apc^**1638**N/+^* (upper) and *Apc^**1638N**^*^/+^*Vdr*^−***/***−^ (lower). Scale bar, 50 μm. (**F**) Comparison of tumor progression at 12 months between *Apc^**1638N**/+^* and *Apc^**1638N**^*^/+^*Vdr*^−***/***−^ mice. **P = 0.0075, two-tailed Chi-Square test. (**G**) Immunodetection of Paneth cells using an anti-lysozyme antibody in sections from the jejunum of each genotype analyzed. Scale bar, 200 μm. (**H)** Staining of Goblet cells using an alcian blue in sections from proximal colon of each genotype analyzed. Shown in G and H are representative images of Paneth and Goblet cell differentiation defect by loss of VDR expression. Scale bar, 200 μm. Data are shown as the mean ± SEM.

**Figure 2:**
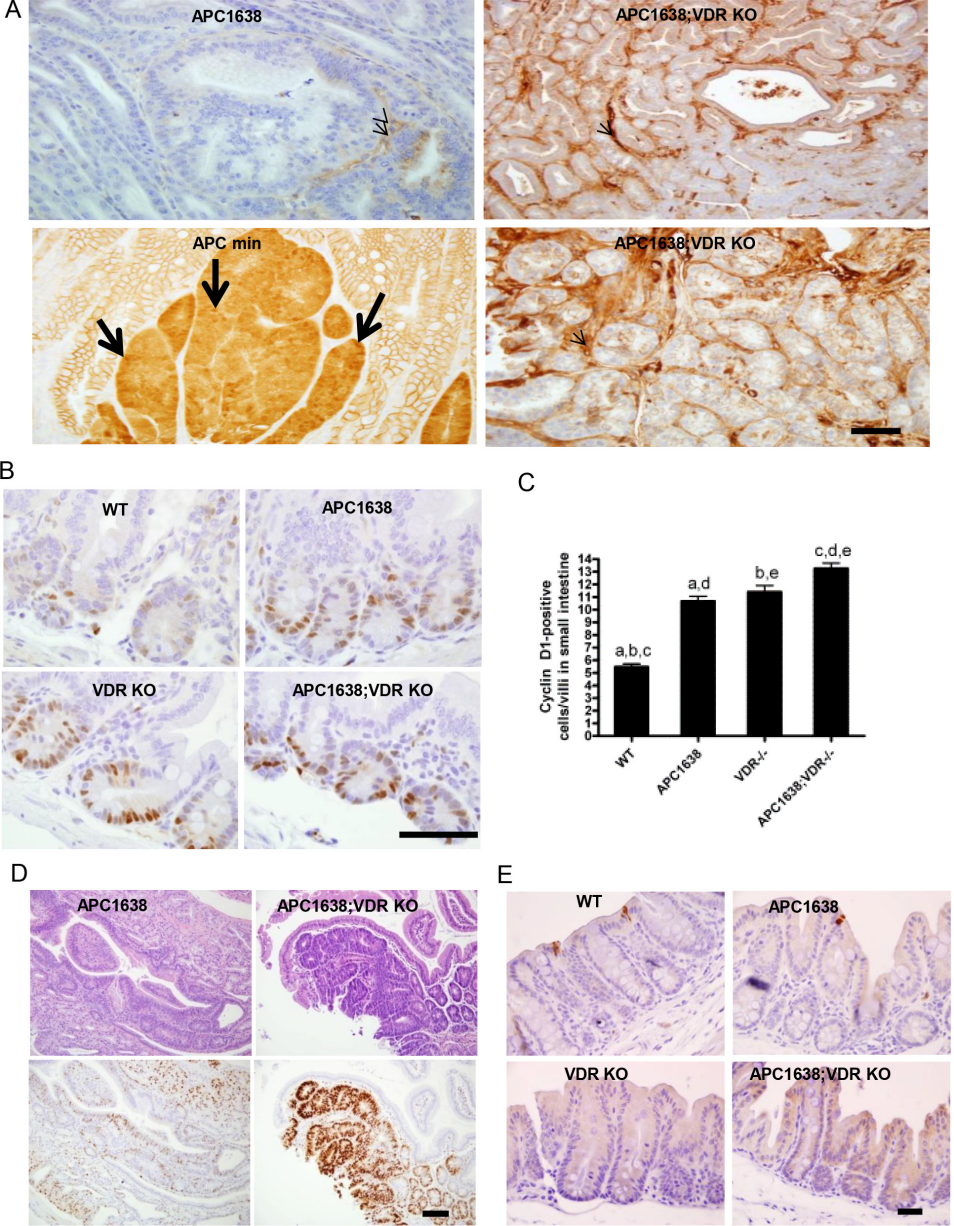
VDR deficiency does not increase β-catenin nuclear localization in tumor sections of *Apc^1638N/+^Vdr^−/−^* mice compared to that of *Apc^min/+^* but significantly activates cyclin D1 gene expression at the crypt base of normal epithelia and tumor cells in *Apc^1638N/+^Vdr^−/−^* mice (**A**) Immunostaining of β-catenin localization using an anti-active β-catenin antibody in tumor sections of *Apc^1638N/+^* (upper left), Apcmin/+ (lower left), and *Apc^1638N/+^Vdr^−/−^*(upper/lower right). Representative images are shown. Arrows indicate β-catenin in the nucleus and arrowheads depict staining of β-catenin in the stroma. Scale bar, 50 μm. (**B**) Immunostaining of cyclin D1, a β-catenin target gene, in normal crypt base epithelial cells of each genotype analyzed. Scale bar, 100 μm. (**C**) Representative images with quantification of cyclin D1 positive cells in sections of normal epithelial cells from each genotype examined. Cells with blue nuclei were considered negative, while cells with brown nuclei were considered positive. The expression of cyclin-D1 was calculated as number of positive cells in 25 complete crypts from at least 3 mice of each genotype. Data are presented as mean ± SEM. ^a^*P* = 0.0001, ^b^*P* = 0.0001, ^c^*P* < 0.0001, ^d^*P* < 0.0001, ^e^*P* = 0.0047 determined by two-tailed *t-test*. (**D**) Representative hematoxylin/eosin staining of tumors derived from *Apc^1638N/+^* (upper left) and *Apc^1638N/+^Vdr*^−*/*−^ mice (upper right) with Immunostaining of cyclin D1 using an antibody in colon tumor sections of *Apc^1638N/+^* (lower left) and *Apc^1638N/+^Vdr*^−*/*−^ mice (lower right). Scale bar, 50 μm. (**E**) Analysis of apoptotic cells by immunostaining of normal large intestinal cells with an anti-active caspase-3 antibody in sections from proximal colon of each genotype analyzed. Representative images are shown. Scale bar, 50 μm.

The observation that vitamin D represses β-catenin signaling and that β-catenin activates the vitamin D receptor raise the possibility that impairment of the vitamin D pathway *in vivo* may affect the onset, incidence or progression of β-catenin-related neoplasias found in APC mutant mice [21]. Studies from other groups used the *Apc*^min/+^*/Vdr*^-−/−^ model to reveal effects of VDR ablation on intestinal tumor size although no differences were observed in tumor latency, incidence, multiplicity, progression or the occurrence of extracolonic lesions [22, 23]. We reasoned that the effects of the *Apc*^min/+^ mutation are so strong that any pro-tumorigenic activity of VDR loss would be difficult to assess. To elucidate the contradictory roles of VDR, we first compared intestinal and non-intestinal lesions in *Apc*^1638N/+^ mice with or without a functional VDR. The hypomorphic *Apc*^1638N/+^ allele provides an attenuated GI cancer phenotype with a higher proportion of large intestinal tumors than the *Apc*^min/+^ model and allows us to examine the effects of manipulations anticipated to exacerbate tumorigenesis [24]. Also unlike *Apc*^min/+^, tumors in the *Apc*^1638N/+^ animals do not exhibit a large increase in the level of β-catenin (unless a ras mutation is also present) allowing for easier analysis of the effects of VDR ablation on this parameter [25]. In contrast to *Apc*^min/+^ our results in the *Apc*^1638N/+^ model indicate that loss of VDR decreased tumor latency in small and large intestine, increased the occurrence of aberrant crypt foci in the proximal large intestine, but did not affect tumor multiplicity regardless of age (Figure 1A–1D). Strikingly, histological analysis showed a marked increase in the proportion of tumors that had progressed to dedifferentiated adenocarcinomas at 12 months (Figure 1E–1F). Consistent with an effect of VDR on differentiation and the regulation of innate immunity, Paneth and Goblet cell differentiation in the unaffected epithelium of the small and large intestine respectively were significantly impaired in *Apc*^1638N/+^*Vdr*^-−/−^ animals (Figure 1G–1H, Supplementary Figure S1), suggesting that loss of the VDR also influences the differentiation of cells involved in innate immunity. As expected in tumors from *Apc*^min/+^ mice staining of activated β-catenin was very intense and throughout the epithelial compartment of the tumor. In contrast but consistent with earlier work very little activated β-catenin is present in *Apc*^1638N/+^ tumors [25]. Remarkably, although increased staining for activated β-catenin occurred in the intestine of *Apc*^1638N/+^*Vdr*^−/−^ mice, this increased staining was largely restricted to the non-epithelial (stromal) compartment. Rarely, activated β-catenin was observed throughout certain epithelial cells, and in some *Apc*^1638N/+^*Vdr*^−/−^ tumor epithelial cells, was found on the basolateral membrane but not nucleus (Figure 2A). However, we did find that levels of the β-catenin target gene cyclin D1 were significantly augmented in normal epithelial and tumor cells upon VDR ablation (Figure 2B–2D). Neither apoptosis (caspase-3 staining) nor cell migration were influenced by VDR ablation (Figure 2E and Supplementary Figure S2). Consequently the decreased tumor latency and increased tumor progression induced by VDR ablation are most likely due to increased cell proliferation via reduced VDR/β-catenin interactions or independently of β-catenin, an observation consistent with the effects of VDR ablation on intestinal hyperplasia [21, 26, 27]. The effects of VDR ablation on ACF in the proximal large intestine (cecum) of *Apc*^1638N/+^ mice is reminiscent, not only of the Gardner syndrome's variant of FAP, but also of inactivation of retinoblastoma protein (Rb) in *Apc*^1638N/+^ mice [28, 29]. Although vitamin D can regulate Rb in other situations no differences in Rb were observed in *Apc*^1638N/+^ or *Apc*^1638N/+^*Vdr*^−/−^ mice [30] (Supplementary Figure S3). Similarly, another vitamin D regulated gene, the cell cycle inhibitor p21, is also decreased in ACF, but we found no differences in *Apc*^1638N/+^ or *Apc*^1638N/+^*Vdr*^−/−^ mice (data not shown) [31].

### Loss of VDR accelerates colon cancer progression and causes anal cancer in azoxymethane treated animals

**Figure 3:**
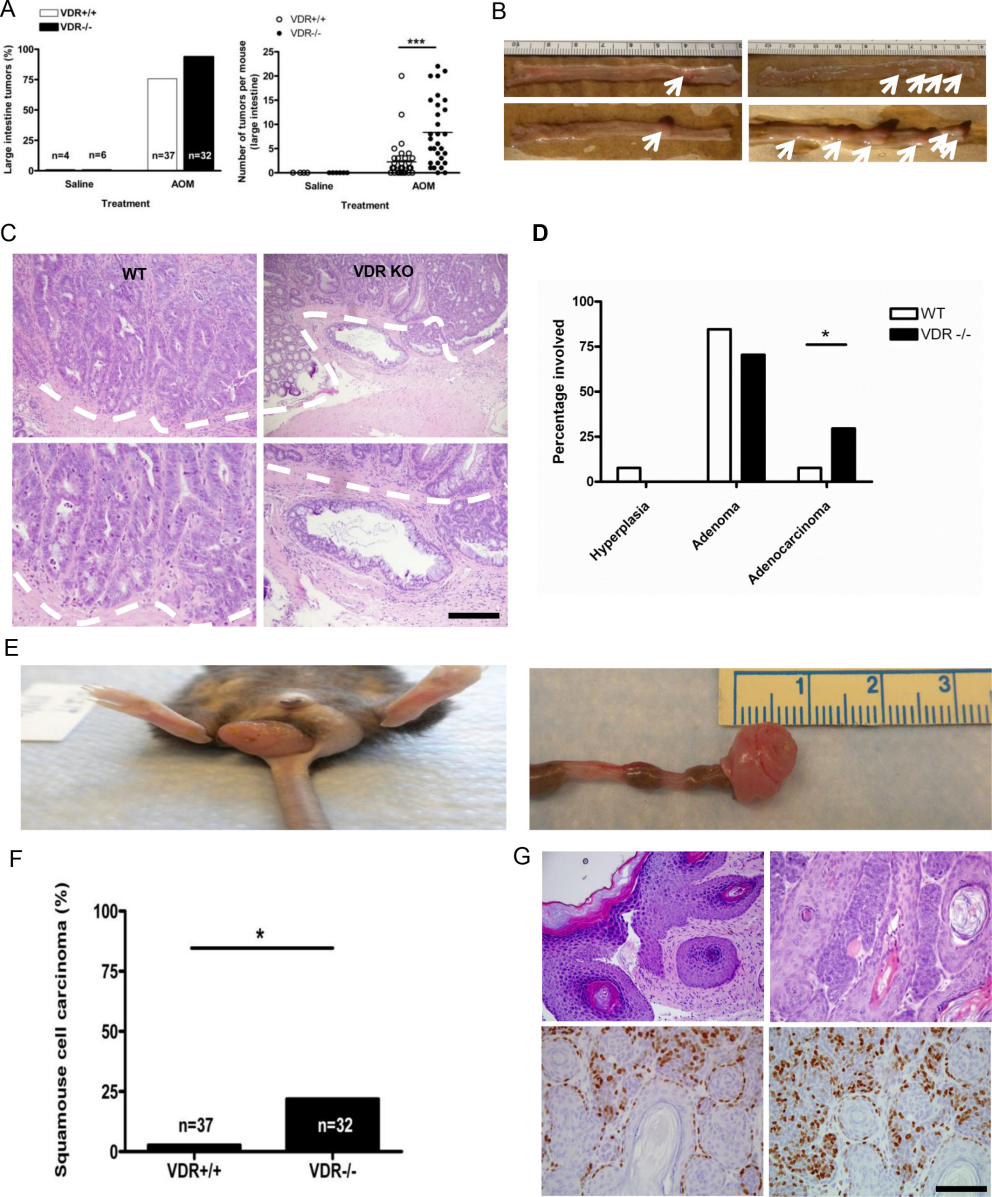
Treatment with azoxymethane does not affect tumor incidence but significantly increases tumor multiplicity in VDR KO mice (**A**) Comparison of intestinal tumor incidence (left) and multiplicity (right) between *Vdr^+/+^* (*n* = 37, tumor multiplicity 2.270 ± 0.6183) and *Vdr*^−*/*−^ (*n* = 32, tumor multiplicity 8.344 ± 1.209 ***P < 0.0001, Two-tailed Student's *t* test) mice. Each dot corresponds to one mouse and the horizontal line indicates the mean. (**B**) Representative images of tumors induced by AOM injection in large intestine from *Vdr^+/+^* (left) and *Vdr*^−*/*−^ (right) mice. White arrows indicates tumor observed in each genotypes. (**C**) Representative hematoxylin/eosin staining images of intestinal tumors from *Vdr^+/+^* (upper left) and *Vdr*^−*/*−^ (upper right) with magnified images in the lower panel. The dashed line demarcates adenocarcinoma observed in VDR KO mice. Scale bar, 100 μm. (**D**) Comparison of tumor progression to adenoma or adenocarcinoma between *Vdr^+/+^* and *Vdr*^−*/*−^ mice (*P < 0.0375, Two-tailed Fisher's test). (**E**) Representative image of squamous cell carcinoma in the anus of *Vdr*^−*/*−^ mice. (**F**) Comparison of squamous cell carcinoma incidence between *Vdr^+/+^*(*n* = 37) and *Vdr*^−*/*−^ mice (*n* = 32 *P < 0.0208 Fisher's exact test). (**G**) Representative hematoxylin/eosin staining images of squamous cell carcinoma from *Vdr*^−*/*−^ (upper panel) and immunostaining of activated β-catenin (lower left) and cyclin D1 in tumor sections (lower right). Scale bar, 100 μm.

We next examined the role of the VDR in colorectal cancer caused by the colon chemical carcinogen, azoxymethane (AOM). AOM commonly induces mutations in both ras and β-catenin and subsequently the development of colorectal but not small intestinal tumors [32]. Because C57BL6/J mice are somewhat resistant to AOM-induced colorectal tumorigenesis compared with other strains, they give us the opportunity to examine the effects of VDR ablation on tumor progression [33]. We treated WT and *Vdr*^−/−^ C57BL6/J with AOM and monitored tumor incidence, multiplicity and progression. Virtually all animals in both groups developed some tumors at 6 months but in contrast to the *Apc*^1638N/+^ model tumor multiplicity was dramatically increased by VDR ablation (Figure 3A–3B). Moreover, examination of the AOM-induced tumors revealed that loss of VDR significantly accelerated tumor progression to adenocarcinoma, implying that VDR functions as tumor suppressor during colorectal tumorigenesis in both tumor models (Figure 3C–3D). Since vitamin D supplementation reduces oxidative DNA damage induced by alkylating agents in the colonic mucosa of patients with colorectal adenoma [34], we hypothesized that an increase of tumor initiation/progression in VDR KO mice may resulted from enhancement of AOM-induced oxidative DNA damage. However, IHC with 8-oxo-dg to specifically detect DNA damage revealed little change among experimental groups (Supplementary Figure S4), implying that rather than increasing DNA damage, loss of VDR amplifies β-catenin/TCF signaling, which in turn leads to increased tumor multiplicity/progression in the distal colon. Interestingly, we observed that *Vdr*^−/−^ mice developed what appeared to be prolapsed colorectal tumors protruding from the anus beginning 3 months after AOM injection (Figure 3E–3F). Subsequent assessment revealed that these lesions were actually squamous cell carcinomas derived from the transitional epithelia/epidermis of the anus (Figure 3G). No lesions were observed in the transitional epithelium at the esophageal/gastric junction, nor elsewhere on the skin. As VDR ablation sensitizes skin to chemically induced squamous cell carcinoma these data suggest that lack of VDR specifically in the transitional epithelia/epidermis of the anus causes susceptibility to chemical carcinogen induced tumorigenesis [35] (Supplementary Figure S5). The anal cancers had increased expression and nuclear localization of activated β-catenin and cyclin D1 consistent with the earlier observation that targeted expression of active β-catenin in the skin of *Vdr*^−/−^ mice induces basal cell carcinoma [36] (Figure 3G). As there are few examples of anal cancer animal models our approach may find utility in preclinical studies of squamous cell carcinoma of the anus [36].

### Loss of VDR causes a high incidence of extracolonic lesions in *Apc^1638N/+^* animals

**Figure 4:**
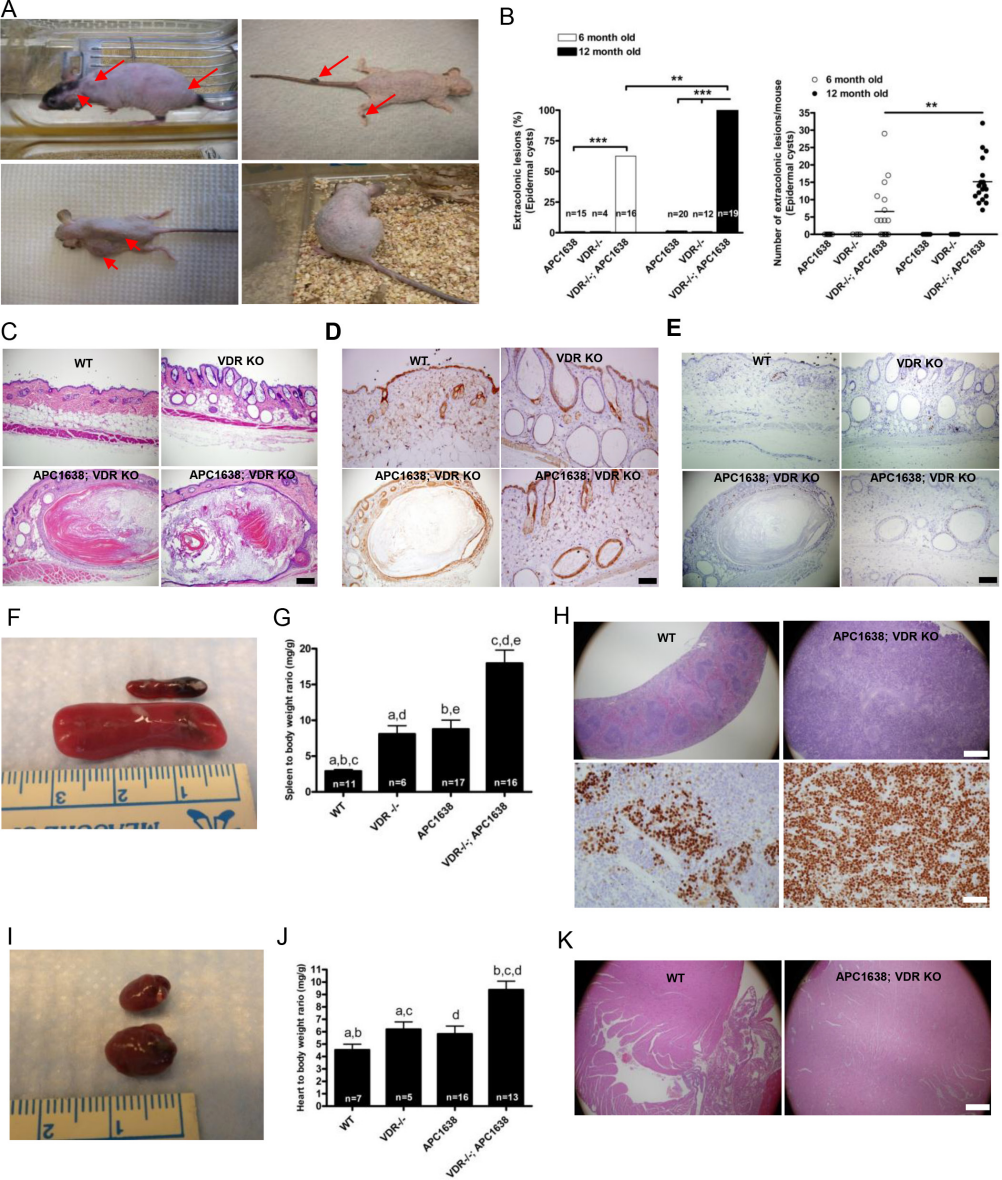
Loss of VDR in *Apc^1638N/+^* mice induces extra-colonic lesions such as epidermoid cysts, splenomegaly, and cardiomegaly (**A**), Representative images of various kinds of skin manifestations such as epidermal cysts, pilomatricoma, fibroma, and pigmented skin lesions in *Apc^1638N/+^Vdr^−/−^* mice. (**B**) Comparison of epidermal cyst incidence (right, **P < 0.0002 and ***P < 0.0001, Fisher's exact test) and multiplicity (left) at age of 6 and 12 months. Each dot (right) represents one mouse examined and the horizontal line indicates the mean (*n* = 16 at 6 months. 6.563 ± 2.041 vs *n* = 19 15.16 ± 1.466, **P < 0.0014, the two-tailed Student's *t-test*). (**C**) Representative hematoxylin/eosin staining images of skin tissues from WT (upper left), *Vdr ^−/ −^* (upper right), and *Apc^1638N/+^Vdr^−/−^* (lower panel), respectively. Scale bar in C, D, and E, 100 μm. (1) Immunostaining of activated β-catenin in skin sections of WT (upper left), *Vdr^−/−^* (upper right), and *Apc^1638N/+^Vdr^−/−^* (lower panel), respectively. (**E**) Immunostaining of cell proliferation using anti-Ki-67 antibody in skin sections of WT (upper left), *Vdr^−/−^* (upper right), and *Apc^1638N/+^Vdr^−/−^* (lower panel), respectively. (**F**) Representative images of spleen in WT and *Vdr^−/−^* mice. (**G**) Comparison of spleen to body weight ratio among different genetic groups. (WT = 11, 2.909 ± 0.2208, *Vdr^−/−^* = 6, 8.085 ± 1.156, *Apc^1638N/+^* = 17, 8.767 ± 1.263 and *Apc^1638N/+^Vdr^−/−^* = 16, 17.97 ± 1.835, ^a^*P* < 0.0001, ^b^*P* = 0.0011, ^c^*P* = 0.0048, ^d^*P* < 0.0001, and ^e^*P* = 0.0002, Two-sided Student's *t-test*). (**H**) Representative hematoxylin/eosin staining of spleen from WT (upper left), and *Apc^1638N/+^Vdr^−/−^* (upper right), respectively. Scale bar, 500 μm. Immunodetection of cell proliferation using anti-Ki-67 antibody in WT (lower left) and *Apc^1638N/+^Vdr ^−/−^* (lower right). Scale bar, 50 μm. (**I**), Representative images of heart from WT and *Apc^1638N/+^Vdr^−/−^* mice. (**J**), Comparison of heart to body weight ratio among different genetic groups. (WT = 7, 4.533 ± 0.4623, *Vdr^−/−^* = 5, 6.194 ± 0.5977, *Apc^1638N/+^* = 16, 5.819 ± 0.6287 and *Apc^1638N/+^Vdr^−/−^* = 13, 9.371 ± 0.7005, ^a^*P* = 0.0494, ^b^*P* = 0.0179, ^c^*P* = 0.0008, and ^d^*P* = 0.0002, Two-sided Student's *t-test*). K, Representative hematoxylin/eosin staining of heart from WT (left), and *Apc^1638N/+^Vdr^−/−^* (right), respectively Scale bar, 500 μm.

Consistent with the notion that VDR might exert an important role in skin by regulating β-catenin signaling, we observed that *Apc*^1638N/+^*Vdr*^−/−^ mice also developed prominent skin lesions. By 12 months, each *Vdr*^−/−^mouse had an average of 15 skin lesions (Figure 4A–4B). We anticipated that the lesions would be tumors, as targeted expression of activated β-catenin results in folliculomas in the skin of *Vdr*^+/+^ mice and basal cell carcinomas in the skin of *Vdr*^−/−^ mice [37]. However when we analyzed skin sections of the *Apc*^1638N/+^*Vdr*^−/−^ mice, we found that most of the lesions were epidermoid cysts or keratocanthomas, filled with keratin (Figure 4C). These skin manifestations are virtually identical to those of patients with the FAP variant Gardner's syndrome [6]. Occasional very small skin lesions and cysts are observed in the *Apc*^1638N/+^ and *Vdr*^−/−^ animals but these are tiny and morphologically quite different. Notably, nuclear β-catenin and Ki-67, a marker for cell proliferation were highly expressed in the epidermal cells surrounding the large cysts in the *Apc*^1638N/+^*Vdr*^−/−^ animals (Figure 4D–4E). Moreover, the presence of epidermal cysts/keratocanthomas prompted us to ask if VDR ablation induced or exacerbated other extra-colonic lesions in *Apc*^1638N/+^animals. Lack of VDR or APC inactivation can induce haematological disorders [38, 39]. Consistent with this we found splenomegaly in both *Vdr*^−/−^ and *Apc*^1638N/+^ animals and spleen to body weight ratio was significantly increased in *Apc*^1638N/+^*Vdr*^−/−^ compared to that of either *Vdr*^−/−^or *Apc*^1638N/+^ animals (Figure 4F–4G). Cell proliferation was markedly increased in the spleen of *Apc*^1638N/+^*Vdr*^−/−^ animals, indicating that deficiency of VDR expression may cause a significant change in myeloid cell differentiation and growth (Figure 4H). Targeted ablation of 1-alpha-hydroxylase or VDR or increased β-catenin signaling leads to cardiac hypertrophy [40, 41]. We also observed cardiomegaly accompanied by cardiomyocyte expansion in *Apc*^1638N/+^*Vdr*^−/−^ animals, suggesting that absence of VDR may cooperate to activate β-catenin signaling, resulting in cardio-hypertrophy (Figure 4I–4K). Differences in tumor progression to adenocarcinoma as well as the incidence of extracolonic lesions in *Apc*^min/+^*Vdr*^−/−^ animals compared to those of *Apc*^1638N/+^*Vdr*^−/−^ animals mouse model strongly suggest that the location of mutation in the APC gene (genotype) and VDR may closely relate to phenotypic changes such as polyp formation and extracolonic lesions in FAP patients, particularly in the context of VDR loss or inactivation.

In order to verify whether the genotype of APC gene may be a potential risk factor for the development of extracolonic manifestations in FAP patients, we first studied the association between genotype and phenotype among FAP patients obtained from the Hereditary Gastrointestinal Cancer Registry (High Risk cancer Clinics, Huntsman Cancer Institute, University of Utah), which includes 457 participants with diagnoses of FAP (Supplementary Table S1). Of the ~198 subjects with verifiable data on APC mutation site and extracolonic lesions, approximately 50% had at least one extracolonic lesion. In those subjects without extracolonic lesions APC mutations were equally distributed among codons 97–1979. However in the 80 subjects with extracolonic lesions APC mutations were far more common in codons 851–1979 than codons 1–850 (Table 1, OR = 6.137; *P* < 0.05). Considering the relatively small number of informative patients this is a highly significant observation. In order to test whether vitamin D pathway genes are related to the Gardner's syndrome, we studied polymorphisms in selected vitamin D pathway genes using blood samples of FAP patients (Supplementary Table S2). Although function disturbing polymorphisms of the VDR gene did not associate with the incidence of epidermal cysts, both A/A and A/C SNPs of vitamin D binding protein (GC protein rs4588) involved in vitamin D transport showed higher odds of extra-colonic lesions including epidermal cysts compared to the control (Supplementary Table S3 and Table 2, OR = 4.82 and 1.922 respectively; *P* < 0.05). In addition, the A/G SNP of the vitamin D metabolizing enzyme (CYP24A) was associated with increased risk of desmoid tumors compared to the control (Supplementary Table S3), suggesting that altered activity of the vitamin D/VDR pathway associated with polymorphisms in genes known to regulate vitamin D activity may be linked to the incidence of extracolonic lesions in FAP patients. Interestingly, while VDR polymorphisms do not associate with either epidermal cysts or desmoid tumors, the Bsm1 Bb SNP of VDR shows a significantly higher risk of other extra-colonic lesions including benign cutaneous symptoms such as fibromas, lipomas and osteomas compared to the control group (Supplementary Table S3 and Table 2, OR = 2.372; *P* < 0.05). In addition less severe truncation APC mutants were often accompanied by GC protein polymorphism (Table 3, OR = 2.031; *P* < 0.05). Several other polymorphisms showed quite a strong trend of association with extracolonic manifestations but these did not quite reach statistical significance (Supplementary Table S3). Taken together, these findings strongly suggest that the polymorphisms of vitamin D/VDR signaling pathway genes, particularly those that co-occur with mutations of APC in codons 851–1979 may be linked to the occurrence of extra-colonic lesions.

**Table 1:** Associations between the extracolonic lesions and location of APC mutations in FAP patients

	Cases with lesions		Cases without lesions			
Lesions	Codon 1–850	Codon 851–1979	Codon 1–850	Codon 851–1979	OR	95% CI
Desmoid tumor	14	19	47	16	3.987	(1.631–9.742)
Epidermal cyst	11	19	62	27	3.966	(1.663–461)
Fibroma	2	8	12	5	9.6	(1.438–62.164)
Lipoma	5	9	10	5	3.6	(0.778–16.662)
Osteomsa	21	29	54	11	6.779	(2.875–15.985)
Combined ALL	37	41	72	13	6.137	(2.931–12.853)

**Table 2:** Associations between SNPs in VDR, GC, CYP24A1 genes and the combined extracolonic lesions risk in FAP patients

SNP	ORs and 95% CI	ORs and 95% CI	ORs and 95% CI	ORs and 95% CI	ORs and 95% CI
Fok1 (rs2228570)	FF vs. ff	Ff vs. ff	FF + Ff vs. ff	FF vs. Ff	FF vs. Ff +ff
	0.471 (0.165–1.344)	0.418 (0.144–1.208)	0.446 (0.162–1.227)	1.152 (0.636–2.078)	0.961 (0.547–1.688)
Bsm1 (rs1544410)	BB vs. bb	Bb vs. bb	BB + Bb vs. bb	BB vs. Bb	BB vs. Bb + bb
	1.387 (0.619–3.107)	0.585 (0.305–1.119)	0.764 (0.418–1.397)	**2.372 (1.108–5.076)**	1.9 (0.934–3.865)
Taq1 (rs731236)	TT vs. tt	Tt vs. tt	TT + Tt vs. tt	TT vs. Tt	TT vs. Tt + tt
	1.042 (0.452–2.404)	0.836 (0.382–1.827)	0.911 (0.434–1.913)	1.247 (0.661–2.355)	1.19 (0.652–2.147)
Apa1 (rs7975232)	AA vs. aa	Aa vs. aa	AA + Aa vs. aa	AA vs. Aa	AA vs. Aa + aa
	0.808 (0.372–1.754)	0.667 (0.351–1.265)	0.71 (0.393–1.283)	1.5 (0.79–2.847)	1.408 (0.78–2.544)
GC Protein (r24588)	G/G vs. A/C	G/G vs. A/A	G/G + A/C vs. A/A	A/C vs. A/A	G/G vs. A/C + A/A
	**1.922 (1.061–3.482)**	2.905 (0.822–10.271)	N.D.	N.D.	N.D.
CYP24A1 (rs2296241)	A/A vs. A/G	A/A vs. G/G	A/A + A/G vs. G/G	A/G vs. G/G	A/A vs. A/G + G/G
	1.492 (0.761–2.925)	1.333 (0.62–2.869)	N.D.	N.D.	N.D.

**Table 3:** Associations between the SNPs in VDR, GC, CYP24A1 and the location of APC mutations in FAP patients

SNP	Codon 1–850		Codon 850–1997	
Fok1(rs2228570)	FF vs. ff	Ff vs. ff	FF vs. Ff	FF vs. Ff
	0.641 (0.332–1.238)	1.729 (0.547–5.466)	1.366 (0.689–2.71)	0.304 (0.664–1.443)
Bsm1(rs1544410)	BB vs. bb	Bb vs. bb	BB vs. bb	BB vs. bb
	0.417(0.183–0.95)	0.807 (0.335–1.946)	1.761 (0.722–4.295)	1.5 (0.578–3.89)
Taq1(rs731236)	TT vs. tt	Tt vs. tt	TT vs. Tt	TT vs. Tt
	0.759 (0.374–1.538)	0.813 (0.325–2.03)	1.292 (0.606–2.756)	1.263 (0.447–3.347)
Apa1(rs7975232)	AA vs. aa	Aa vs. aa	AA vs. Aa	AA vs. Aa
	0.652 (0.321–1.324)	1.01 (0.433–2.357)	1.563 (0.731–3.339)	1.37 (0.554–3.385)
GC Protein(r24588)	G/G vs. A/C	G/G vs. A/A	G/G vs. A/G	A/C vs. A/A
	0.475 (0.246–0.917)	0.175 (0.034–0.891)	**2.031 (1.021–4.04)**	1.391 (0.321–6.024)
CYP24A1(rs2296241)	A/A vs. A/G	A/A vs. G/G	A/A vs. A/G	A/G vs. G/G
	0.692 (0.323–1.483)	1.333 (0.62–2.869)	1.667(0.72–3.858)	1.759 (0.709–4.36)

## DISCUSSION

Our genetic and chemical-induced tumor models show that in addition to accelerating gastrointestinal tumor progression somatic loss of the VDR or activity disturbing SNPs in vitamin D pathway genes results in extra-colonic lesions that model the Gardner's syndrome form of FAP (on an APC mutant background) and anal cancer (following carcinogen administration).

It is well known that cyclin D1 is required for gastrointestinal tumorigenesis in the APC^min^ model [42]. Consistent with this, VDR depletion in both APC^min^ and APC^1638^ models alters cyclin D1 expression in the colonic epithelium (the present study and [22, 23]. However, in contrast to the APC^min^ model, VDR manipulation in the hypomorphic APC^1638^ model did not result in changes in total β-catenin levels or its localization in the colonic epithelium. Although cyclin D1 is clearly a β-catenin target gene, the common cyclin D1 changes in both models, implies the following: 1. Change in the level of β-catenin is not the major mechanism for VDR-mediated transcriptional regulation of the cyclin D1 gene in the APC^1638^ model, 2. That VDR directly regulates the activity of existing β-catenin in the nucleus in the APC^1638^ model, or, 3) the effects of VDR on cyclin D1 are not mediated by changes in β-catenin level, localization or activity but rather by another VDR-regulated transactivator. In this regard vitamin D is known to alter the expression of other genes that have the capacity to alter cyclin D1 transcription and/or activity [43].

Our finding that the less severely truncating APC mutations were much more likely to lead to extracolonic manifestations is somewhat counter-intuitive and lead us to examine more closely the relationship of mutation site to APC protein partner binding [44]. Several recent studies have revealed new functions and partners for APC and show important roles for α-catenin and Hippo/Yap pathway regulation in addition to modulation of β-catenin stability [45] (Supplementary Figure S6). α-catenin binding to APC is now known to be required for APC to form a stable interaction with β-catenin and promote its degradation. α-catenin also modulates the activity of the Hippo/Yap pathway, which in turn results in tissue overgrowth and tumors in multiple organ types but classically resulting in early onset keratocanthomas (epidermoid cysts) and cardiomyocyte hyper proliferation [46]. Since both of these phenotypes were observed in FAP patients with less severe APC truncations and/or the *Apc*^1638N/+^*Vdr*^−/−^ mouse our data points to a role for APC mutation site in the differential regulation of the Hippo/Yap and β-catenin pathways. At this time it is not known which region of APC beyond codon 850 is actually responsible for regulation of Hippo/Yap signaling but it is quite plausible that some APC mutants found in FAP patients could regulate both Yap and β-catenin, others only Yap, and others only β-catenin [47]. If less severely truncated mutants of APC are associated with extracolonic lesions in humans with FAP it is curious that extracolonic manifestations only occur (keratocanthomas) or are potentiated (cardiomyocyte proliferation) in the *Apc*^1638N/+^ mice when VDR is absent. Unlike laboratory mice, which are vitamin D replete, most human populations, particular those that are chronically ill and/or have deficits in intestinal function, such as the FAP cohort in the present study, are often significantly vitamin D deficient [48]. Although we do not have data on vitamin D levels in the Utah cohort it is highly likely that they are vitamin D deficient. Taken together these observations strongly implicate the vitamin D pathway as a potential modifier in Gardner's syndrome. However, it is not likely that severe function perturbing mutations of the vitamin D receptor in human are associated with Gardner's syndrome as rickets has not been reported in FAP patients. Our data indicate that altered activity of the vitamin D/VDR pathway associated with polymorphisms in genes that regulate vitamin D transport and/or metabolism may contribute to the occurrence of the extra-colonic manifestations in FAP patients. In addition, regulation of the vitamin D/VDR axis in the context of other co-factors such as mutation or viral infection may also be important in the etiology of anal cancer, a rapidly growing public health problem with no viable preclinical models [36].

## MATERIALS AND METHODS

### Animal studies

All animal studies were approved by the institutional animal care committee. Mice were maintained in a virus- and parasite-free animal facility under a 12-h light, 12-h dark cycle. *Apc*^1638N/+^ mice on a C57BL/6 background were purchased from Jackson Laboratory (Bar Harbor, Maine). *Vdr*^+/−^ and *Vdr*^−/−^ mice on a C57BL/6 background have been reported previously [49]. *Apc*^1638N/+^*Vdr*^−/−^ mice were produced by crossing *Apc*^1638N/+^ × *Vdr*^+/−^. Mouse genotyping was performed by genomic PCR. All animals were fed with standard rodent chow diet except VDR-deficient mice that were fed with diet containing 2% calcium, 1.25% phosphorus, and 20% lactose supplemented with 2.2 IU vitamin D/g (TD96348, Teklad, Madison, WI) from 21 days of age [50]. Animals were sacrificed at 6 and 12 months for analysis. 24 hours before sacrifice, three mice from WT and VDR KO were injected i.p. with 50 mg/kg BrdU to label proliferating crypt cells. For chemical carcinogen-induced tumorigenesis study, at 6 weeks of age, WT (*n* = 41) and VDR KO (*n* = 38) mice were randomly divided into a control and AOM treatment group. Mice were injected i.p. with AOM (10 mg/kg body weight) once a week for 6 weeks in a vehicle of normal saline [51]. Control animals (6 from each strain) received vehicle alone. Mice were sacrificed 6 months after the first injection. After sacrifice, the entire intestine was dissected, cut longitudinally, placed onto a filter paper with luminal side facing up and then fixed flat in 10% formaldehyde (made in phosphate buffered saline [PBS], pH 7.2). The number of visible polyps and tumors in the intestine was counted and macroscopic tumor tissues were paraffin-embeded and sections were cut at 4μm with a Leica microtome 2030. Slides were deparaffinized, hydrated and stained with hematoxylin and eosin (H&E). Histological evaluation was performed according to the intestinal tumor classification of the rodent [52].

### Histology and immunostaining

Immunohistochemical staining was performed for β-catenin (1:50, Transduction labs), Brdu(1:150, Cell Signaling1:), caspase-3(1:250, Abcam), cyclin-D1(1:50, Neomarker), Ki-67(1:12, DAKO), lysozyme(1:60, Pierce), 8-oxo-dG(1:100, Gene Tex), and Rb(1:750, Santa Cruz Biotechnology). Four micron sections from formalin fixed paraffin embedded tissues were de-paraffinized with xylenes and rehydrated through a graded alcohol series. Heat induced epitope retrieval (HIER) was performed by immersing the tissue sections at 98°C for 20 minutes in 10 mM citrate buffer (pH 6.0) with 0.05% Tween. Immunohistochemical staining was performed using the VectaStain Kit from Vector Labs according to manufacturer's instructions. Briefly, slides were treated with 3% hydrogen peroxide for 10 minutes. Endogenous biotin was blocked using an avidin/biotin blocking kit from Invitrogen. The slides were then treated with 10% normal serum and exposed to primary antibodies for 1 hour at room temperature. Slides were exposed to appropriate biotin-conjugated secondary antibodies (Vector Labs), Vectastain ABC reagent and DAB chromagen (Dako). Slides were counterstained with Hematoxylin (Fisher, Harris Modified Hematoxylin) at a 1:17 dilution for 2 minutes at RT, blued in 1% ammonium hydroxide for 1 minute at room temperature, dehydrated, and mounted with Acrymount. Consecutive sections with the omitted primary antibody were used as negative controls.

For Alcian blue histochemistry, staining was performed according to:

http://www.nottingham.ac.uk/pathology/protocols/alcblue.html

### Detection of ACF and colon tumors

The colons were resected and gently flushed with 10% neutralized formalin to remove residual bowel contents, cut open longitudinally, fixed flat between filter papers, and submerged in 10% neutralized formalin overnight at 4°C. Fixed colons were stained with 0.2% methylene blue, as described [50]. The numbers of ACF were counted for each mouse. ACF were identified as lesions composed of enlarged crypts, with an increased pericryptal area, slightly elevated appearance above the surrounding mucosa with an oval or slit-like orifice, and higher staining intensity with 0.2% methylene blue than normal crypts.

### Study population and genotyping

The Hereditary Gastrointestinal Cancer Registry (High Risk Cancer Clinics, Huntsman Cancer Institute, University of Utah) includes 604 participants (539 of whom are still living) with diagnose of FAP or AFAP (attenuated familial adenomatous polyposis) in 181 kindreds, who are linked to the Utah Population Database. 604 participants have an IRB-approval protocol that allow them to electronically query the medical records warehouse for discrete diagnose among the deidentified Utah population. Genotyping was performed on blood-derived genomic DNA using six individual TaqMan SNP Genotyping Assays (Applied Biosystems #4351376 (VDR and GC) and #4332072 (CYP24A1)). Assay IDs were as follows: VDR (rs2228570) C__12060045_20, VDR (rs1544410) C___8716062_10, VDR(rs731236) C___2404008_10, VDR (rs7975232) C___28977635, GC (rs4588) C___8278879_10, CYP24A1 (r22296241) AHRSPW3 (forward primer: CGTGGCCTCTTTCATCACAGA reverse primer: TTTTGCGGTTGTTTTCTTTGAAGGT probe: TGCCCATAAAATCG/AGCCAA). Assays were performed with TaqMan Genotyping Master Mix (Applied Biosystems #4371357) following manufacturer's recommended conditions on a Bio-Rad CFX96 Real-Time PCR System. Alleles were assigned to each sample using Bio-Rad CFX Manager software.

### Statistical analysis

Data was analyzed using GraphPad Prism 5.0 biochemical statistical package (GraphPad Software, Inc., San Diego, CA). Values of all measurements were expressed as mean ± SEM. Statistical analysis was performed using two-tailed unpaired student *t-test* for continuous variables or Fisher's exact test for categorical variables. *P-value* < 0.05 was considered significant at the significance level of 0.05. For association studies with SNPs, logistic regression and multinomial logistic regression models are used.

## SUPPLEMENTARY MATERIALS


