# Supplementary material for: A role for the vitamin D pathway in non-intestinal lesions in genetic and carcinogen models of colorectal cancer and in familial adenomatous polyposis
Source: Oncotarget. 2016 Oct 19;7(49):80508–20. doi: 10.18632/oncotarget.12768 (PMC5348337; doi:10.18632/oncotarget.12768)
Supplement: Supplementary file 1 [file oncotarget-07-80508-s001.pdf]

A role for the vitamin D pathway in non-intestinal lesions in genetic and carcinogen models of colorectal cancer and in familial adenomatous polyposis

Supplementary Materials

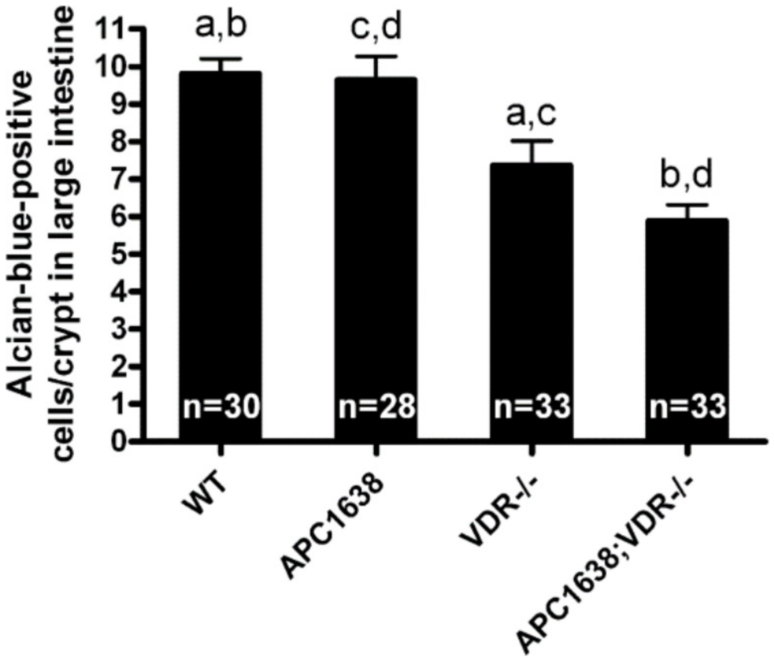

Supplementary Figure S1: Loss of VDR expression impairs Goblet cell differentiation in *Apc*$^{1638N/+}$ mice. Large intestinal tissue sections from each genotypic group were stained with Alcian blue, a marker for Goblet cell differentiation, which were expressed as number of Alcian blue positive cells in the 25 full large epithelial cells from at least 3 mice of each genotype. WT, *Apc*$^{1638N/+}$, *Vdr*$^{-/-}$, and *Apc*$^{1638N/+}$ *Vdr*$^{-/-}$, $^{a}P = 0.0029$, $^{b}P < 0.0001$, $^{c}P = 0.0152$, and $^{d}P < 0.0001$,Two-tailed *t*-test.

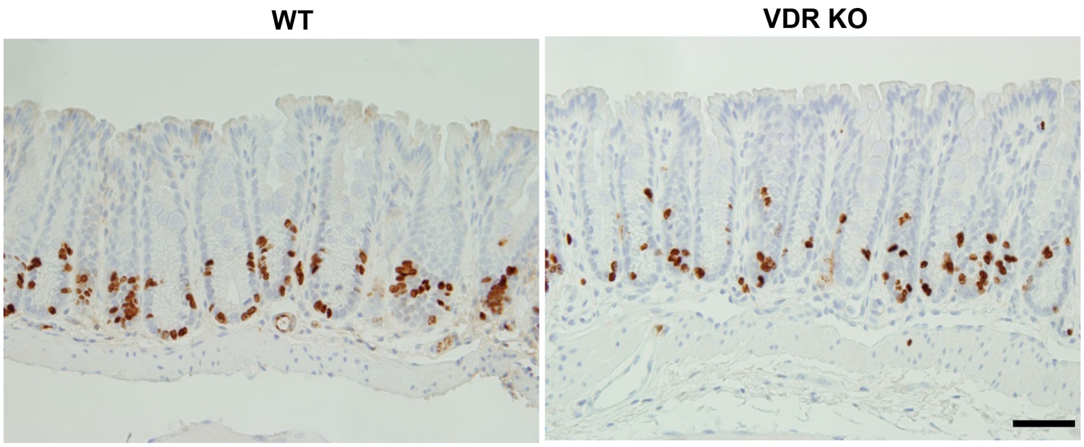

Supplementary Figure S2: VDR does not affect cell migration. Brdu was intraperitoneally injected into WT and VDR KO mice for 4 hours before sacrificing mice and large intestinal tissue section was immunostained using anti-Brdu antibody to reveal cell migration. Scale bar, 50μm.

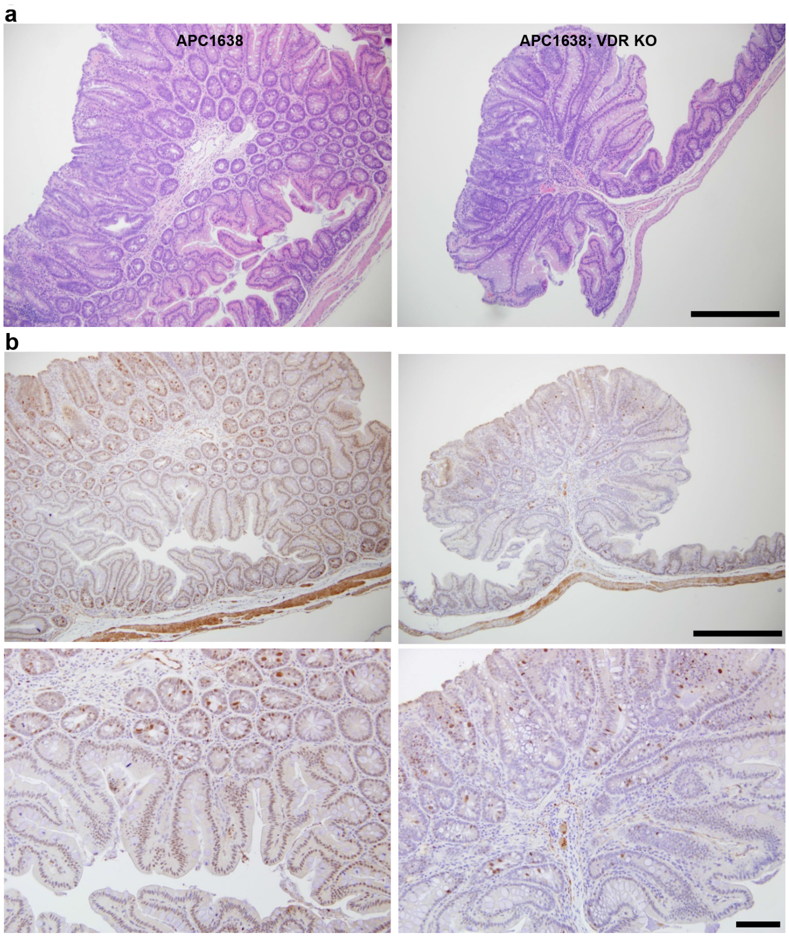

Supplementary Figure S3: Phosphorylation of Rb was not changed in the absence of functional VDR expression in Apc1638N/+ mice. (**a**) Representative hematoxylin/eosin staining images of tumor tissue section from *Apc*$^{1638N/+}$ (upper left) and *Apc*$^{1638N/+}$ *Vdr*$^{-/-}$ (upper right), Scale bar, 50 µm. Immunostaining of phosphorylated Rb in *Apc*$^{1638N/+}$ (lower left) and *Apc*$^{1638N/+}$ *Vdr*$^{-/-}$ (lower right). Scale bar in (**b**) upper, 200µm and (b) lower, 50µm respectively.

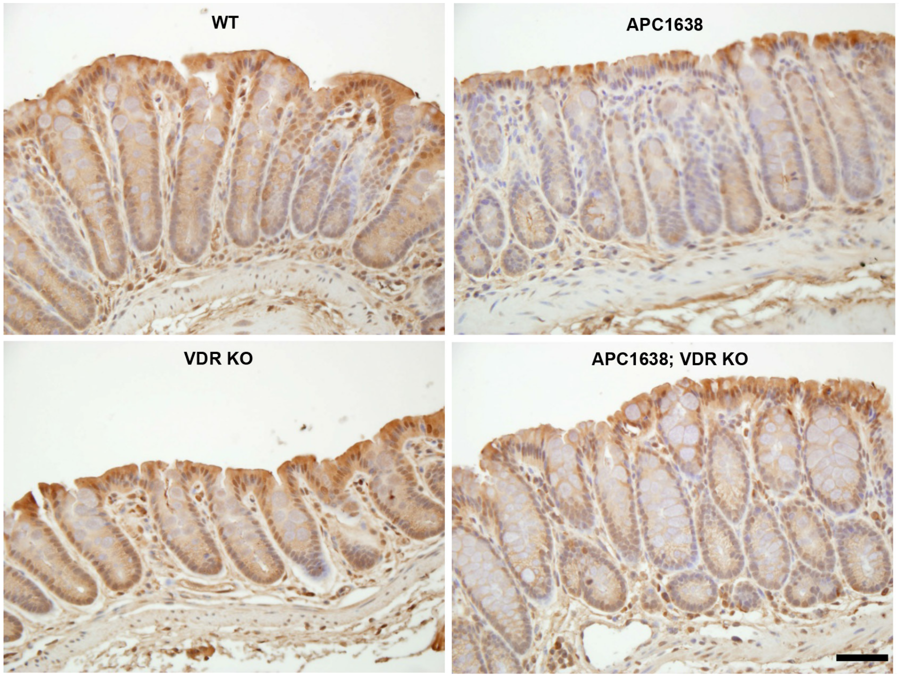

Supplementary Figure S4: AOM does not induce DNA damage regardless of functional expression of VDR. DNA damage was detected using anti-8-oxo-dG antibody from the large intestinal tissue sections of each genotype group. Scale bar, 50 µm.

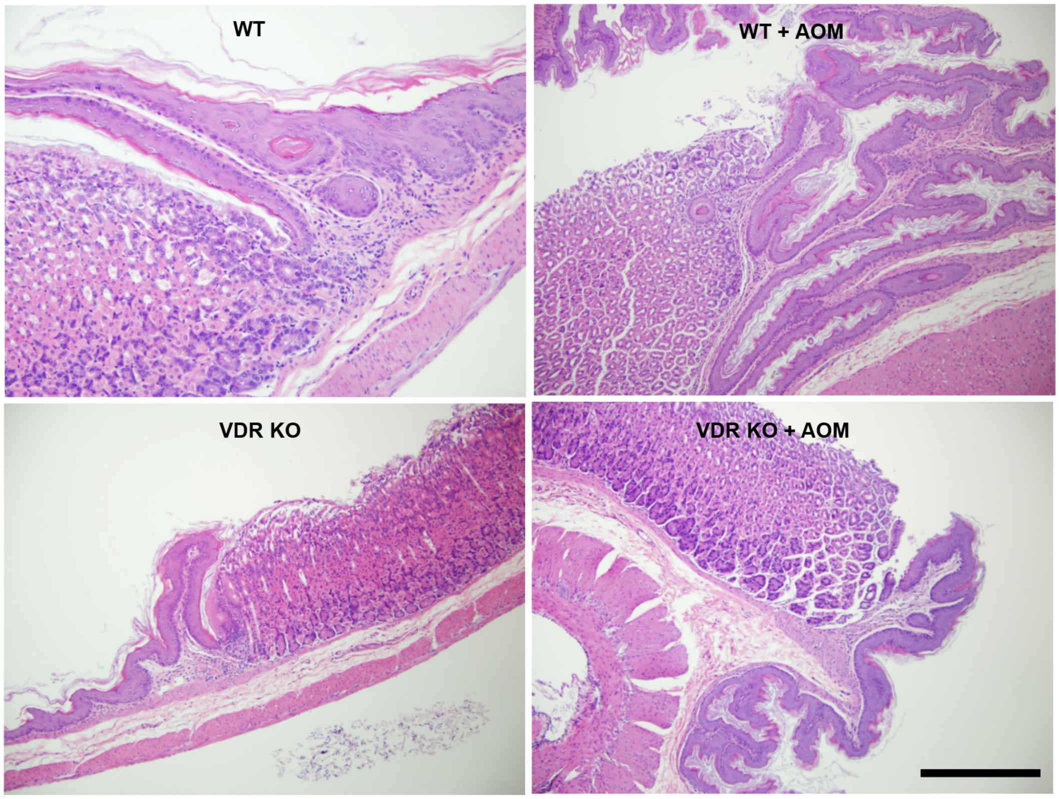

Supplementary Figure S5: Treatment with AOM does not affect the transitional epithelium at the gastric junction in the absence of VDR. Representative hematoxylin/eosin staining images of transitional epithelium at the gastric from WT (upper left), WT treated with AOM (upper right), *Vdr*$^{-/-}$ (lower left), and *Vdr*$^{-/-}$ treated with AOM (lower right). Scale bar, 200 µm.

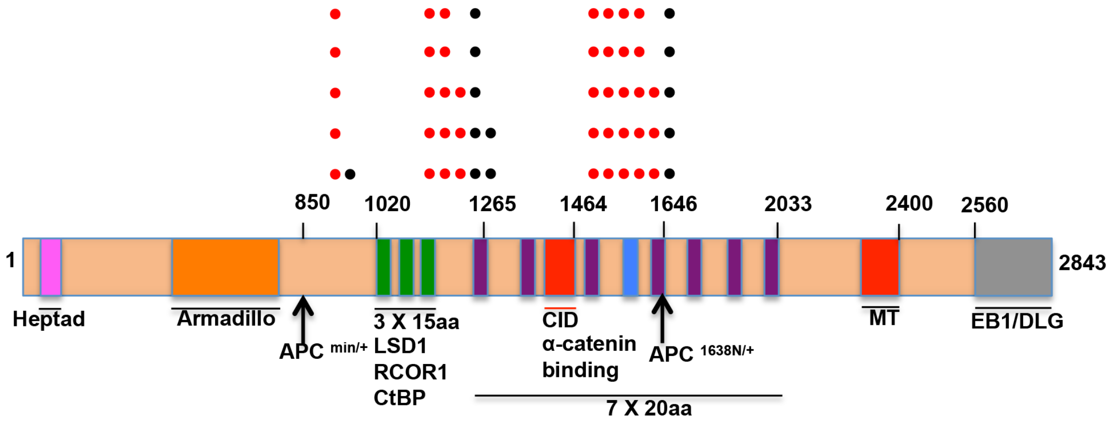

Supplementary Figure S6: Schematic diagram of the APC gene with relative risk of developing extracolonic lesions corresponding to APC mutation site indicated as the red dots while the black dots indicate cases without extracolonic lesions.

Supplementary Table S1: Genotype and allele frequencies of VDR, GC and CYP24A1 gene polymorphisms and location of APC mutations in FAP patients

SNP	Cases (n=457)	Genders		APC Mutations	
VDR		Male	Female	Codon 1-850	Codon 851-1997
Fok1(rs2228570)					
FF	211	89	122	146	37
fF	194	85	109	143	33
ff	52	20	32	39	5
Bsm1(rs1544410)					
BB	86	32	54	67	13
bB	204	89	115	136	40
bb	167	73	94	125	22
Taq1(rs731236)					
TT	170	74	96	130	20
TC	215	90	125	145	41
CC	72	30	42	53	14
Apa1(rs7975232)					
AA	134	57	77	92	28
CA	213	89	124	151	32
CC	110	48	62	85	15
GC protein(rs4588)					
G/G	253	111	142	194	34
A/C	172	67	105	108	38
A/A	33	17	16	27	3
CYP24A1(r2296241)					
A/A	144	58	86	114	14
A/G	222	95	127	153	38
G/G	92	42	50	62	23

Supplementary Table S2: Distribution of SNPs in VDR, GC and CYP24A1 genes in FAP patients with extracolonic lesions

SNP	Lesions											
VDR	Epidermalcyst		Desmoid		Osteoma		Lipoma		Fibroma		Combined	
Fok1 (rs2228570)	Cases	Control	Cases	Control	Cases	Control	Cases	Control	Cases	Control	Cases	Control
FF	21	41	21	45	29	44	9	6	5	7	47	48
Ff	16	43	22	24	24	29	7	6	6	7	42	38
ff	0	13	3	8	8	4	0	3	0	4	6	13
Bsm1 (rs1544410)												
BB	4	18	9	19	9	23	3	3	1	5	15	26
Bb	18	49	25	34	34	33	6	5	6	5	52	38
bb	15	30	12	24	18	21	7	7	4	8	28	35
Taq1 (rs731236)												
TT	16	29	12	25	19	22	7	7	4	7	29	34
TC	17	54	25	36	32	38	5	6	6	8	50	47
CC	4	14	9	16	10	17	4	2	1	3	16	18
Apa1 (rs7975232)												
AA	8	36	13	36	19	34	4	5	1	9	30	39
CA	18	39	23	31	29	31	5	4	8	3	45	39
CC	11	22	10	10	13	12	7	6	2	6	20	21
GC protein (rs4588)												
G/G	14	54	18	44	27	44	9	9	2	11	42	61
A/C	18	39	24	29	28	30	6	6	8	7	45	34
A/A	5	4	4	4	6	3	1	0	1	0	8	4
CYP24A1 (rs2296241)												
A/A	5	26	7	27	15	28	5	2	2	5	24	32
A/G	22	43	26	33	28	33	4	6	6	5	47	42
G/G	10	28	13	17	18	16	7	7	3	8	25	25

Supplementary Table S3: Associations between SNPs in VDR, GC, CYP24A1 genes and the individual extracolonic manifestation risk in FAP patients

	ORs and 95% CI	ORs and 95% CI	ORs and 95% CI	ORs and 95% CI	ORs and 95% CI	Lesions
Fok1 (rs2228570)	FF vs. ff	Ff vs. ff	FF + Ff vs. ff	FF vs. Ff	FF vs. Ff +ff	
	0.3(0.062-1.457)	0.413(0.084-2.039)	0.349(0.075-1.625)	0.726(0.334-1.582)	0.558(0.26-1.199)	Epidermoid cyst
	0.804(0.193-3.339)	0.409(0.096-1.74)	0.602(0.151-2.393)	1.964(0.904-4.27)	1.674(0.802-3.496)	Desmoid
	3.03(0.84-11.01)	N.D.	2.755(0.788-9.627)	1.26(0.61-2.57)	1.471(0.749-2.891)	Osteoma
	0.056-3.583)	N.D.	0.5(0.072-3.477)	0.778(0.713-3.493)	0.519(0.124-2.165)	Lipoma
	0.7(0.09-5.432)	N.D.	0.636(0.098-4.138)	1.2(0.246-5.844)	0.764(0.167-3.487)	Fibroma
Bsm1 (rs1544410)	BB vs. bb	Bb vs. bb	BB + Bb vs. bb	BB vs. Bb	BB vs. Bb + bb	
	2.25(0.646-7.839)	1.361(0.598-3.097)	1.523(0.695-3.338)	1.653(0.493-5.547)	1.88(0.591-5.979)	Epidermoid cyst
	1.056(0.368-3.026)	0.68(0.287-1.614)	0.779(0.345-1.762)	1.552(0.602-3.999)	1.347(0.551-3.292)	Desmoid
	2.19(0.81-5.93)	N.D.	1.116(0.53-2.35)	**2.63(1.06-6.52)**	2.461(1.042-5.813)	Osteoma
	1.00(0.418-6.722)	N.D.	0.889(0.216-3.66)	1.20(0.164-8.80)	1.083(0.182-6.439)	Lipoma
	2.5(0.214-29.256)	N.D.	0.714(0.153-3.334)	6.00(0.516-69.757)	3.846(0.386-38.344)	Fibroma
Taq1 (rs731236)	TT vs. tt	Tt vs. tt	TT + Tt vs. tt	TT vs. Tt	TT vs. Tt + tt	
	0.518(0.146-1.84)	0.908(0.263-3.129)	0.719(0.22-2.344)	0.571(0.252-1.293)	0.56(0.256-1.224)	Epidermoid cyst
	1.172(0.403-3.41)	0.81(0.309-2.122)	0.927(0.372-2.311)	1.447(0.614-3.408)	1.362(0.604-3.071)	Desmoid
	0.681(0.252-1.893)	N.D.	0.692(0.291-1.645)	0.975(0.45-2.113)	0.884(0.425-1.841)	Osteoma
	2.00(0.272-14.7)	N.D.	2.167(0.334-14.05)	0.833(0.171-4.058)	1.125(0.273-4.635)	Lipoma
	0.583(0.044-7.661)	N.D.	0.5(0.045-5.512)	1.312(0.259-6.643)	1.114(0.236-5.255)	Fibroma
Apa1 (rs7975232)	AA vs. aa	Aa vs. aa	AA + Aa vs. aa	AA vs. Aa	AA vs. Aa + aa	
	2.25(0.784-6.455)	1.083(0.434-2.703)	1.442(0.616-3.375)	2.077(0.805-5.36)	2.139(0.883-5.181)	Epidermoid cyst
	0.361(0.122-1.065)	0.487(0.212-1.119)	0.449(0.205-0.981)	2.055(0.894-4.724)	**2.229(1.019-4.875)**	Desmoid
	1.939(0.793-5.087)	N.D.	1.467(0.615-3.497)	1.674(0.786-3.565)	1.748(0.864-3.535)	Osteoma
	1.458(0.264-8.048)	N.D.	1.167(0.279-4.871)	1.562(0.243-10.031)	1.5(0.315-7.137)	Lipoma
	3.00(0.220-40.928)	N.D.	0.444(0.072-2.74)	24.00(2.060-279.597)	10(1.05-95.199)	Fibroma
GC Protein(rs4588)	G/G vs. A/C	G/G vs. A/A	G/G + A/C vs. A/A	A/C vs. A/A	G/G vs. A/C + A/A	
	1.78(0.79-4.01)	**4.82(1.14-20.35)**	N.D.	N.D.	N.D.	Epidermoid cyst
	2.023(0.936-4.37)	2.444(0.551-10.851)	N.D.	N.D.	N.D.	Desmoid
	1.521(0.753-3.073)	3.259(0.752-14.125)	2.691(0.644-11.235)	N.D.	1.679(0.853-3.306)	Osteomas
	1.0(0.232-4.31)	0.5(0.038-6.547)	0.5(0.041-6.121)	N.D.	1.167(0.279-4.871)	Lipoma
	6.286(1.022-38.652)	2.750(0.162-46.794)	0.9(0.072-11.207)	N.D.	7.071(1.167-42.847)	Fibroma
CYP24A1(rs2296241)	A/A vs. A/G	A/A vs. G/G	A/A + A/G vs. G/G	A/G vs. G/G	A/A vs. A/G + G/G	
	2.66(0.9-7.88)	1.86(0.56-6.16)	N.D.	N.D.	N.D.	Epidermoid cyst
	**3.039(1.144-8.076)**	2.95(0.98-8.87)	N.D.	N.D.	N.D.	Desmoid
	1.584(0.709-3.540)	2.10(0.837-5.271)	1.596(0.733-3.467)	N.D.	1.752(0.832-3.692)	Osteoma
	0.267(0.034-2.116)	0.4(0.057-2.80)	0.469(0.093-2.369)	N.D.	1.731(0.273-10.975)	Lipoma
	3.0(0.396-22.712)	0.938(0.114-7.729)	0.469(0.093-2.369)	N.D.	1.731(0.273-10.975)	Fibroma
